# Developmental delay in a resource-constrained environment: An approach to early intervention

**DOI:** 10.4102/safp.v63i1.5355

**Published:** 2021-08-31

**Authors:** Pragashnie Govender, Vasantha Govender, Deshini Naidoo

**Affiliations:** 1 Discipline of Occupational Therapy, College of Health Sciences, University of KwaZulu-Natal, Durban, South Africa; 2 Department of Paediatric Neurology, KwaZulu-Natal Children’s Hospital, Durban, South Africa; 3 Department of Paediatric Neurology, Inkosi Albert Luthuli Central Hospital, Durban, South Africa

**Keywords:** developmental delay, paediatric intervention, primary healthcare, resource constrained, multidisciplinary team

## Abstract

With a reduction in mortality rates of children under 5 years, in low- and middle-income countries, the responsibility to provide quality care to the increased number of surviving children becomes essential. Many of these children present with developmental delay and the onus inevitably rest on the healthcare system. There is, therefore, the need for recognising timely intervention as routine care for these children, who may have potential for a better quality of life with intervention. The authors advocate for early referral and intervention, and provide a brief overview of a holistic approach to developmental delay in low resourced settings from their perspective.

## Introduction

The timely and accurate assessment of a child’s development can serve to detect developmental delays (DDs) earlier in childhood^1^ and aid in focused early intervention.^2^ The recent re-emphasis on early intervention for children with DD in low- and middle-income countries^3^ calls for action that is contextually situated and relevant. In this study, the authors describe considerations for a multisystemic approach to promoting early intervention for DD.

## Describing the context in which early intervention is located

From a global perspective, the World Health Organization (WHO), United Nations International Children’s Emergency Fund and World Bank have proposed the Nurturing Care Framework to promote child health and well-being, and advocate for optimal developmental outcomes for a child.^4^

From a national level, we are aware that the national Department of Health (DoH) has prioritised health in early childhood as part of the national service delivery areas (NSDAs) to reduce maternal and child mortality.^5^ The integrated school health policy of 2012 is one of the key national DoH components proposed to strengthen the delivery of primary healthcare (PHC) service in order to promote childhood development and reduce adverse events amongst children.^6^ Furthermore, the Framework and Strategy for Disability and Rehabilitation services^7^ outline the intention for early identification of disability and DD through screening and community mapping, school health outreach teams and health promotion programmes, aimed at stimulating developmental outcomes at post-natal and well-baby clinics. Similarly, the Department of Social Development (DSD) National Integrated Early Childhood Policy,^8^ and more recently, the amendment of the South African Schools Act through the Basic Education Laws Amendment for mandatory attendance at learning centres 2 years prior to grade 1^9^ emphasises the national government’s espoused commitment to early intervention. There is thus the need for integrating these services for early intervention.

**FIGURE 1:**
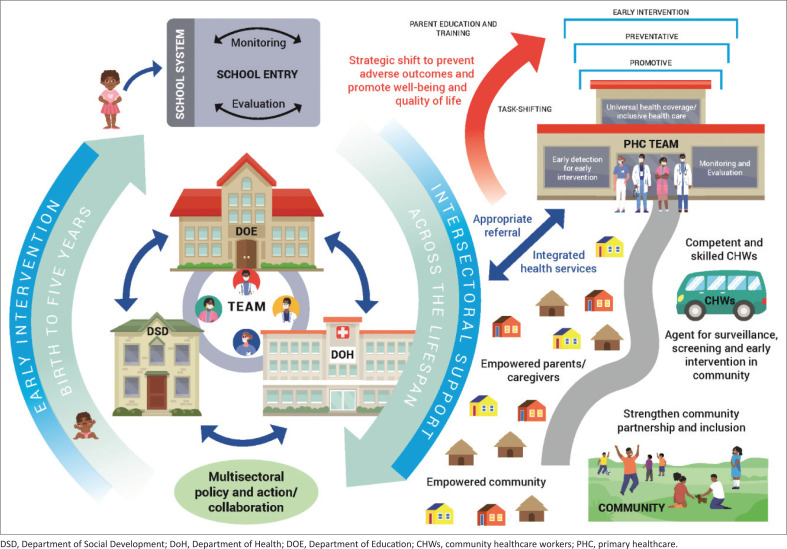
Context of service provision in a low-resourced context of South Africa.

Access to care can occur at a community level. At a PHC level, we aim for universal health coverage, and acknowledge this as a suitable point for identifying and addressing the needs of children with DD, who are often hidden in their communities.^4^ Whilst some care is possible at the PHC level, referrals to the various levels of the healthcare system (district–regional–tertiary) would occur depending on the underlying aetiology and diagnostic workup required.^1^ Provision of specialised services may also occur outside of the PHC setting. With this, we have the interaction of the DSD (with early childhood development programmes) and department of education (DOE) (responsible for monitoring of child who enters the school system) interfacing with the health system. Acknowledging these structures in the holistic care of the child with DD is essential, as access to services and supports should not be fragmented but rather enhanced for maximal benefit to the child and family (Figure 1).

## Benefit of integration of services

The DSD and DoH are key role players in early intervention prior to the child entering grade R. There is the need for ensuring that services are co-ordinated for optimal use of limited resources. For example, both the DSD and DoH have community healthcare worker (CHWs) and both departments offer early intervention. If services were coordinated, resources could be pooled and the service could be enhanced by more frequent screening by the CHWs. The provision of specialised care by the ward outreach team and community service therapists as part of early intervention could also be maximised in this manner. This would create a value-added service as there is potential to reduce the risk of DD, as screening would allow for children at risk to receive intervention earlier and surveillance of children who already present with DD would occur. Moreover, because of the low numbers of specialised services in the low-resourced areas, there is a need for task shifting, especially amongst the different habilitation therapists and the CHWs. This may allow for more comprehensive services to be offered by the habilitation therapists.

## Promoting early intervention at primary healthcare level

There is a need for a strategic shift by healthcare workers to prevent adverse outcomes and promote well-being and quality of life of children with DD. In order to facilitate this shift within low-resourced contexts, the following requires consideration:

- Appropriate screening, surveillance and assessment for early identification with relevant tools for DD in low to middle income countries (LMICs).^1,10^
- Prevention of DD should include interventions in the first 1000 days to improve well-being; both physical and mental health of pregnant mothers from conception, by education and training of caregivers, and continual screening and surveillance of infants at all clinic visits.
- Access to appropriate services, including habilitation, prevention (e.g. screening), health promotion (e.g. workshops for parents), and facilitating access and the ability to sustain educational opportunities. This would include access to relevant healthcare workers, such as nurses, doctors, occupational therapists, speech therapists, physiotherapists, dieticians, audiologists and psychologists. The team may need to work in transdisciplinary roles, given the limited human resources of specialised staffing.
- Social determinants of health, such as poverty, lack of access to nutrition, parental influences and the environment, when planning or providing early intervention
- Strengthening community participation and inclusion
- Directing early intervention that aims to empower parents, caregivers and the community
- Ensuring competent and skilled CHWs as agents for early detection, referral and intervention
- Facilitation of multi-sectoral policy action or collaboration between health, social development and education ministries
- Advocating and ensuring integrated healthcare services at PHC, district or regional and tertiary levels
- Monitoring and evaluation at the various stages (at 0–5 years at PHC) and DOE (5 years onwards at school entry).

**FIGURE 2:**
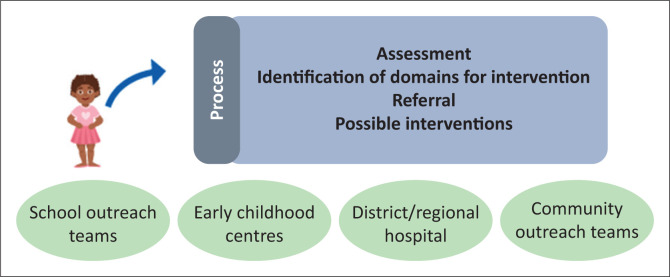
Process within the intervention context.

When considering early intervention, the continuum of care begins with prevention of DD with interventions geared at women in their pregnancy, education on simple measures to enhance parent–child interactions in the early developmental period, and then assessment of the child with the health professional recognising the intervention required, according to the domains of functioning at that point in time. This may be followed by appropriate referral at multiple points across childhood with many members of the multidisciplinary team within the variable intervention context (Figure 2). We suggest that the continuum of care begin at the district-level hospital or the CHC where prenatal care is provided and where the child is born. It would be important to ensure that neonate is assessed and the mother is offered education on normal development whilst in recovery post-delivery.

Table 1 provides an overview of the possible strategies against some key considerations that have been highlighted in the available literature.

**TABLE 1:** Promoting early intervention at a primary healthcare level.

Key areas for consideration		Possible strategies
**PHC contex**t	Utilisation of the Road to Heath Care Chart is ineffective in rural districts for under-5 child health management^11^	To *review the tools* used and promote use of appropriate instruments or tests within a PHC context. Govender et al.^1^ and Faruk et al.^10^ in a systematic review highlight possible screening tools or tests for LMIC contexts. *• Further education* required for PHC workers
	Development screening as part of well-baby clinics regularly lack the knowledge to correctly identify and refer children with DD^12^	
	Need for strategic co-ordination for integrated care at pivot points^13^	*• Developing partnerships* with civil society (e.g. faith-based organisations, advocacy groups, non-governmental and non-profit organisations, early child development centres) Developing *stronger links* with early learning programmes
**The multidisciplinary team**	When children are identified, the availability of early intervention services are often limited in resource-poor settings^14^	Creating *standard operating processes* for identification, referral, and follow up of children with DD and at risk for DD within specific contexts *Interdisciplinary approaches* where possible may be suitable, in this context, to address all domains of functioning. MDT to facilitate extension for *collaboration multi-sectorally* MDT *task shifting to CHWs* where possible for interventions that need to reach homesteads. MDT should take responsibility for *facilitating access to quality care and integrating intervention and strengthening MDT collaboration* for holistic management, case management, follow up and further referral (to other levels of care) *Discipline-specific interventions* should be offered where possible CHWs extending their role to community education to work ***towards reducing the stigma*** attached to DD and childhood disability Strengthening **supervision and support** for CHWs within their roles in surveillance and EI for DD.
	Early intervention and multidisciplinary care remain critical in potentially allaying the loss of a child’s development potential^15^	
**Upskilling of CHWs**	CHWs expressed the need for more appropriate training and upskilling to manage childhood conditions more appropriately^16^	CHWs to serve as the intermediary in surveillance and referral *Education and training of CHWs* on early signs and symptoms of DD or red flags^1^ Skilling to provide parent education on ***s****trategies for responsive caregiving* Skilling to provide parent education on *strategies for promoting early learning* Using the vehicle of Phila Mtwana (healthy baby) programme for DD *surveillance, referral and monitoring.*
**Caregiver or parent education**	Limited knowledge on early childhood development and in providing age-appropriate stimulation, and limited knowledge on services available for DD^17^	Parent education on *responsive caregiving in the first 3-years of life* and parent strategies for *home-based activities* that can assist development across domains This is with the understanding that early nurturing home environments protect young children against effects of early adversities^23^ and interventions in the first 3 years of life improved early child cognitive, language, motor, socioemotional development, and attachment and reduced behavioural problems^24^ Strategies for *baby-bonding and attachment* that can be executed in the home setting, for example, infant massage.^25^ Including *content on caregiver burden, maternal mental health* and how to maintain the *quality of life for caregivers* of children with DD *Parent workshops* on how to make and use low-cost stimulation toys or apparatus for developmental stimulation in the home Strategies on how to *integrate developmental stimulation* into everyday tasks, routines and activities
	Early evaluation and intervention include not only management and treatment for the individual child but also provision of appropriate family and community-based support mechanisms^18^	
	Parents of children with DD may be more likely to have low-quality parent-child interactions because of increased difficulty in parenting this population^19^	
	Understanding what families’ needs are and how families use and integrate strategies within the context of their daily lives provides practitioners with insights needed to support families’ resiliency in promoting their children’s participation^20^	
	Parents could be intervention agents to facilitate their children’s language and social communication outcomes^21^ with parent-implemented interventions at time proving more effective than clinician-directed service provision^22^	

CHWs, community healthcare workers; DD, developmental delays; PHC, primary health care; EI, early intervention; MDT, multidisciplinary team; LMIC, low to middle-income country/ies.

## Conclusion

A child with DD may present unique challenges to practitioners at various levels. With a current focus in many countries to transcend beyond child survival to support optimal development, the onus strongly rests on service providers within multidisciplinary teams at all levels of care to ensure that these children realise their potential towards becoming contributing members of the society. Within low-resourced contexts, service delivery that promotes well-being is reliant on early identification, timely and appropriate early intervention, and well-coordinated and affordable care. In this study, we have provided an overall perspective on how this may be realised in a low-resourced context, although applicability may go across childhood disabilities.

## References

[1] Govender V, Naidoo D, Govender P. Developmental delay in a resource-constrained environment: Screening, surveillance and diagnostic assessment. S Afr Fam Pract Off J S Afr Acad Fam Pract Prim Care. 2021;63(1):e1–e4. 10.4102/safp.v63i1.5306 PMC8378019 34082557

[2] Milbrath G, Constance C, Ogendi A, Plews-Ogan J. Comparing two early child development assessment tools in rural Limpopo, South Africa. BMC Pediatr. 2020;20:197. 10.1186/s12887-020-02101-0 32380968 PMC7204218

[3] Smythe T, Zuurmond M, Tann CJ, Gladstone M, Kuper H. Early intervention for children with developmental disabilities in low and middle-income countries – The case for action. Int Health [serial online]. 2021 [2021 Aug 1];13(3):222–231. Available from: https://academic.oup.com/inthealth/article/13/3/222/5891235. 10.1093/inthealth/ihaa044 PMC8079317 32780826

[4] World Health Organization, United Nations Children’s Fund, World Bank Group. Nurturing care for early childhood development: A framework for helping children survive and thrive to transform health and human potential. Geneva: World Health Organization; 2018.

[5] South African Department of Health and Basic Health. Integrated School Health Policy [homepage on the Internet]. Pretoria; 2012 [2021 Aug 2]. Available from: https://serve.mg.co.za/content/documents/2017/06/14/integratedschoolhealthpolicydbeanddoh.pdf

[6] South African Department of Health. Delivery agreement for outcome 2: A long and healthy life for all South Africans [homepage on the Internet] [2021 June 30]. Available from: https://www.gov.za/sites/default/files/delivery%20agreement%20Health%20Sector%20NSDA.pdf

[7] South African Department of Social Development. 2015 National Integrated Early Childhood Development Policy [homepage on the Internet] [2021 Aug 2]. Pretoria. Available from: https://www.gov.za/sites/default/files/gcis_document/201610/national-integrated-ecd-policy-web-version-final-01-08-2016a.pdf

[8] National Department of Health. Framework and strategy for disability and rehabilitation service in South Africa 2015–2019. Pretoria; 2014.

[9] South African Government. Draft basic education laws Amendment Bill [homepage on the Internet] [2021 June 30]. Available from: https://www.education.gov.za/Resources/Legislation/CallforComments/DraftBELABill.aspx

[10] Faruk T, King C, Muhit M, et al. Screening tools for early identification of children with developmental delay in low-and middle-income countries: A systematic review. BMJ Open. 2020;10(11):e038182. 10.1136/bmjopen-2020-038182 PMC7684835 33234622

[11] Win T, Mlambo MG. Road-to-Health Booklet assessment and completion challenges by nurses in rural primary healthcare facilities in South Africa. S Afr J Child Health. 2020;14(3):124. 10.7196/sajch.2020.v14i3.01685

[12] Van der Merwe MN, Mosca R, Swanepoel DW, Glascoe FP, Van der Linde J. Early detection of developmental delays in vulnerable children by community care workers using an mHealth tool. Early Child Dev Care. 2019;189(5):855–866. 10.1080/03004430.2018.1480481

[13] Slemming W, Saloojee H. Beyond survival: The role of health care in promoting ECD. S Afr Child Gauge. 2013:50–55.

[14] Kyarkanaye T, Dada S, Samuels AE. Collaboration in early childhood intervention services in Gauteng. Infants Young Child. 2017;30(3):238–254. 10.1097/IYC.0000000000000095

[15] Ogundele MO. A multidisciplinary approach to the assessment and management of pre-school age neuro-developmental disorders: A local experience. J Nurs Care Pract. 2017;1:1–2. 10.1097/IYC.0000000000000095

[16] Naidoo S, Naidoo D, Govender P. Community healthcare worker response to childhood disorders: Inadequacies and needs. Afr J Prim Health Care Fam Med. 2019;11(1):1–0. 10.4102/phcfm.v11i1.1871 PMC6556913 31038346

[17] Ayob Z, Christopher C, Naidoo D. Exploring caregivers’ perceptions on their role in promoting early childhood development. Early Child Dev Care. 2021:1–5. 10.1080/03004430.2021.1888943

[18] Trude AC, Richter LM, Behrman JR, Stein AD, Menezes AM, Black MM. Effects of responsive caregiving and learning opportunities during pre-school ages on the association of early adversities and adolescent human capital: An analysis of birth cohorts in two middle-income countries. Lancet Child Adolesc Health. 2021;5(1):37–46. 10.1016/S2352-4642(20)30309-6 33340466 PMC7763480

[19] Te Kaat-Van den Os DJ, Jongmans MJ, Volman MC, Lauteslager PE. Parent-implemented language interventions for children with a developmental delay: A systematic review. J Policy Pract Intellect Dis. 2017;14(2):129–137. 10.1111/jppi.12181

[20] Coussens M, Vitse F, Desoete A, Vanderstraeten G, Van Waelvelde H, Van de Velde D. Participation of young children with developmental disabilities: Parental needs and strategies, a qualitative thematic analysis. BMJ Open. 2021;11(4):e042732. 10.1136/bmjopen-2020-042732 PMC8021744 33795296

[21] Akamoglu Y, Meadan H. Parent-implemented language and communication interventions for children with developmental delays and disabilities: A scoping review. Rev J Autism Dev Dis. 2018;5(3):294–309. 10.1007/s40489-018-0140-x

[22] DeVeney SL, Hagaman JL, Bjornsen AL. Parent-implemented versus clinician-directed interventions for late-talking toddlers: A systematic review of the literature. Commun Dis Q. 2017;39(1):293–302. 10.1177/1525740117705116

[23] Jeong J, Franchett EE, Ramos de Oliveira CV, Rehmani K, Yousafzai AK. Parenting interventions to promote early child development in the first three years of life: A global systematic review and meta-analysis. PLoS Med. 2021;18(5):e1003602. 10.1371/journal.pmed.1003602 33970913 PMC8109838

[24] Scherzer AL, Chhagan M, Kauchali S, Susser E. Global perspective on early diagnosis and intervention for children with developmental delays and disabilities. Dev Med Child Neurol. 2012;54(12):1079–1084. 10.1371/journal.pmed.1003602 22803576 PMC3840420

[25] Perks LM, Rencken G, Govender P. Therapists’ consensus on an infant massage programme for high-risk infants from resource constrained contexts: A Delphi study. S Afr J Occup Ther. 2020;50(3):72–82. 10.17159/2310-3833/2020/vol50no3a9

